# Supplementary figures and images for: Inducible and reversible inhibition of miRNA-mediated gene repression in vivo
Source: eLife. 2021 Aug 31;10:e70948. doi: 10.7554/eLife.70948 (PMC8476124; doi:10.7554/eLife.70948)

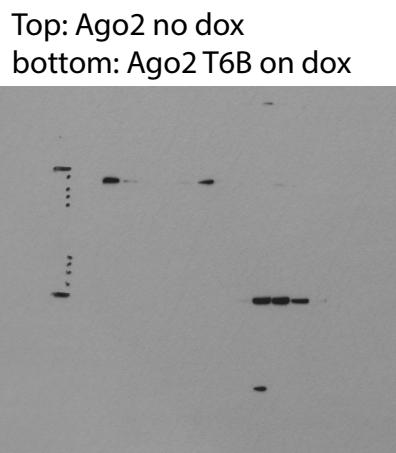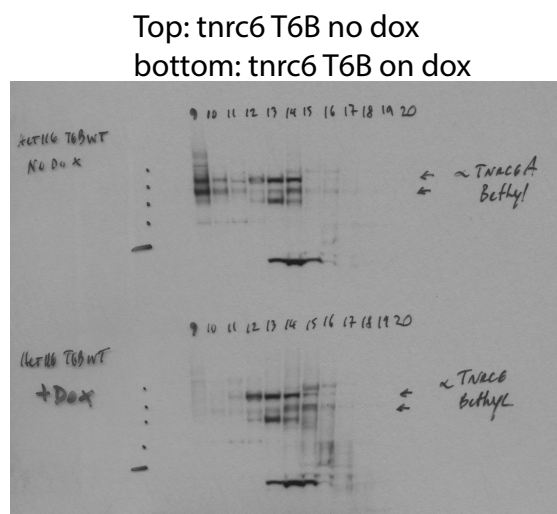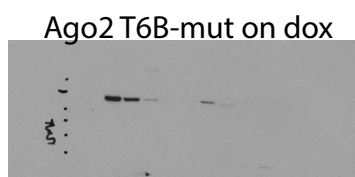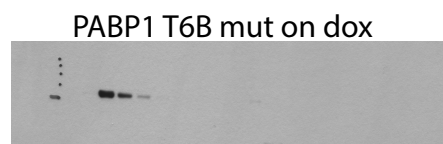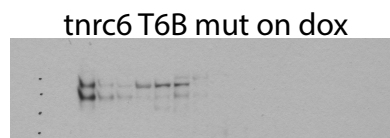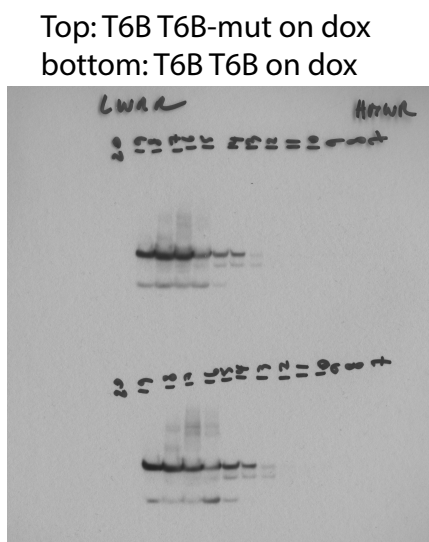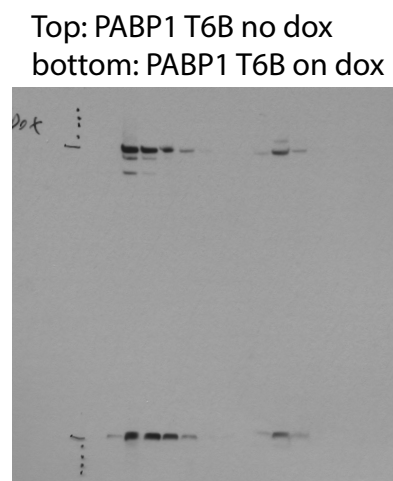

Figure 1-source data 4. Unedited gels Figure 1C.

Supplement: Figure 1—source data 4. [file elife-70948-fig1-data4.pdf]

GAPDH

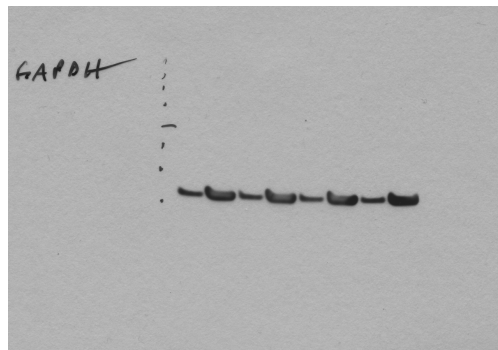

T6B

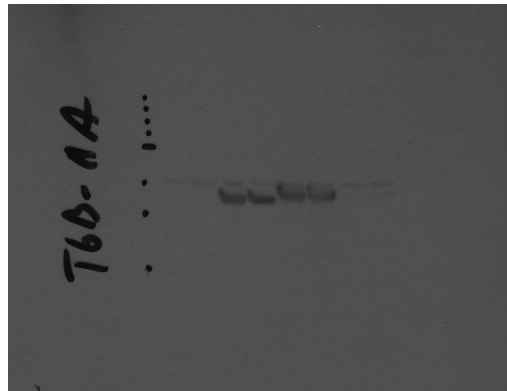

Tubulin

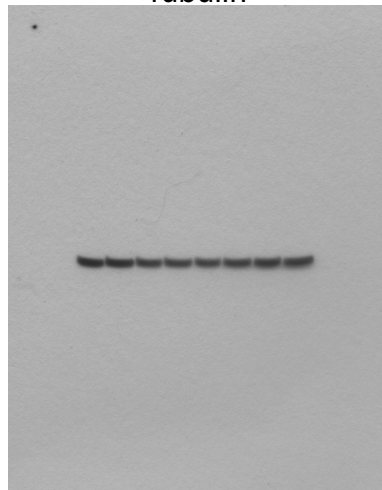

Figure 1-source data 6. Unedited gels used in Figure 1F.

Supplement: Figure 1—source data 6. [file elife-70948-fig1-data6.pdf]

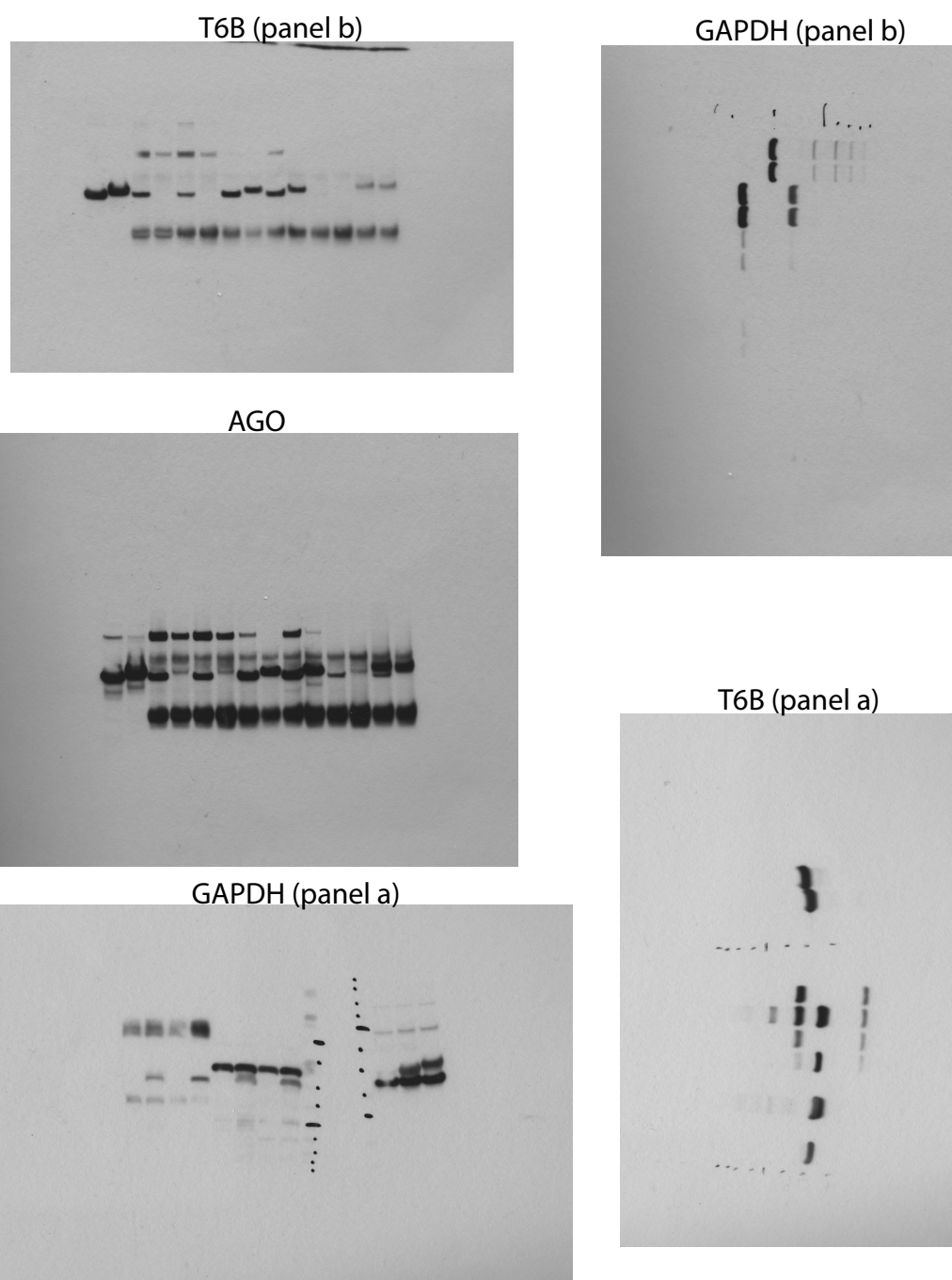

**Figure 1-figure supplement 1-source data 1**-Unedited blots shown in Figure 1-figure supplement 1

Supplement: Figure 1—figure supplement 1—source data 1. [file elife-70948-fig1-figsupp1-data1.pdf]

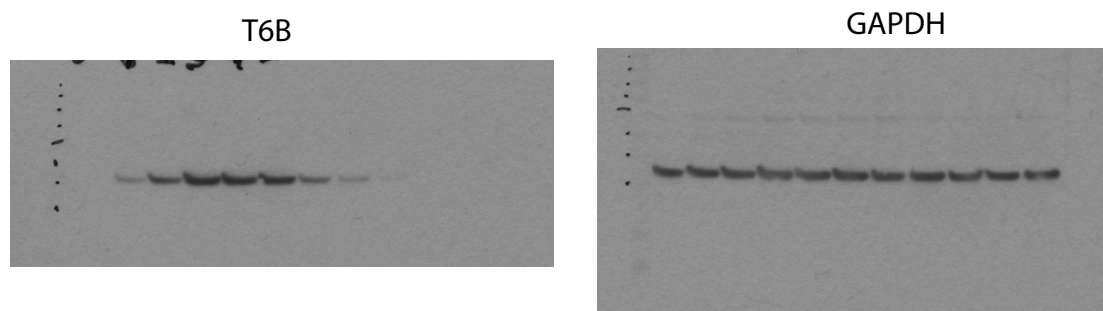

**Figure 2-source data 3. Unedited blots shown in Figure 2C.**

Supplement: Figure 2—source data 3. [file elife-70948-fig2-data3.pdf]

AGO2

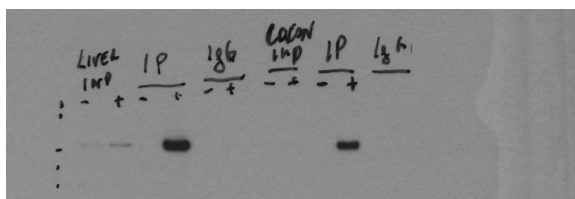

GAPDH

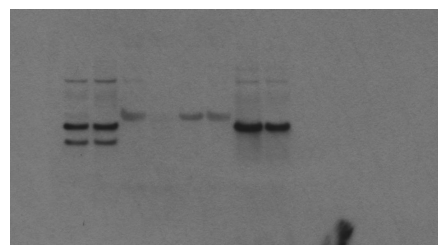

T6B

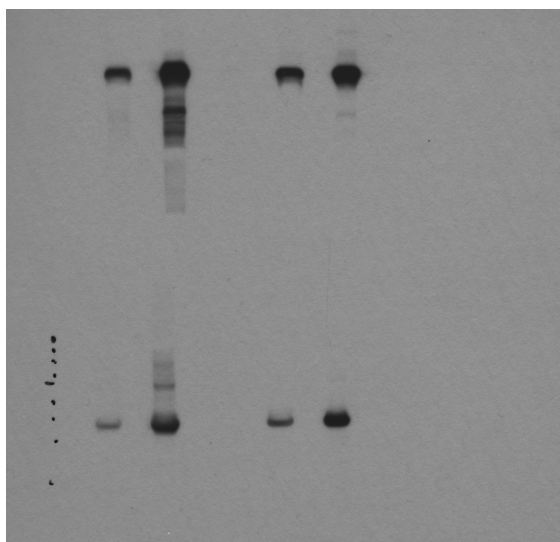

Figure 2-source data 5. Unedited blots shown in Figure 2D.

Supplement: Figure 2—source data 5. [file elife-70948-fig2-data5.pdf]

AGO2 Day 0

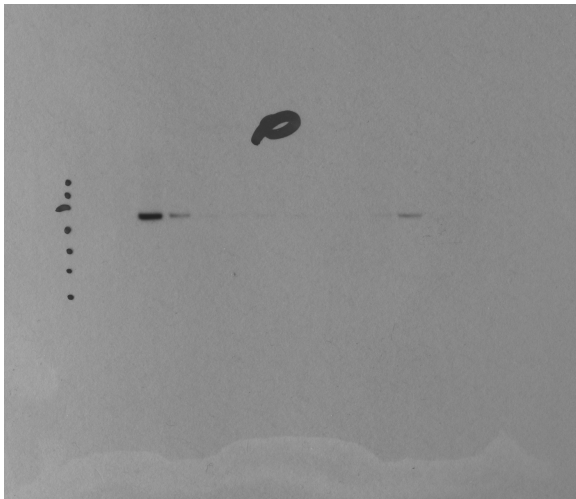

AGO2 Day 5

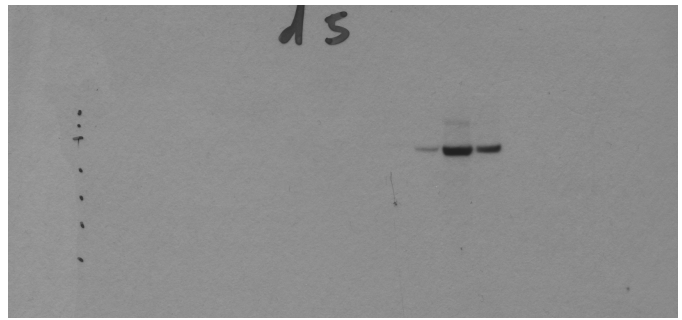

AGO2 Day 5 off dox

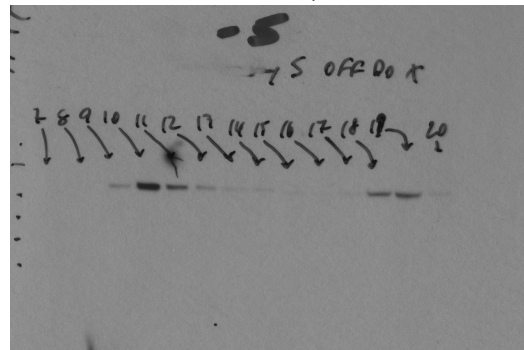

Figure 2-source data 7. Unedited blots shown in Figure 2E

Supplement: Figure 2—source data 7. [file elife-70948-fig2-data7.pdf]

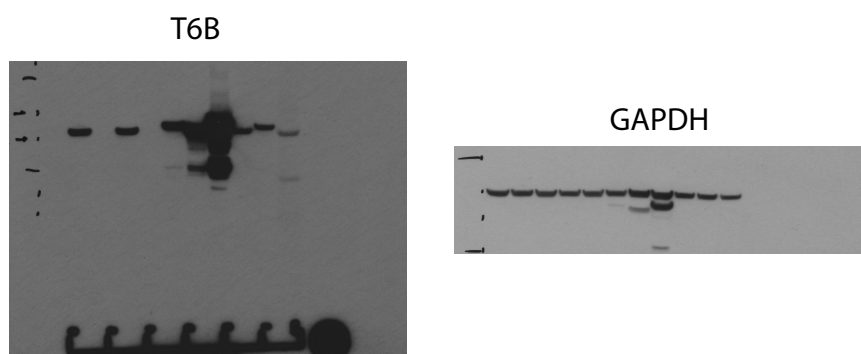

**Figure 2-figure supplement 1-source data 1.**Unedited blots shown in Figure 2-figure supplement 1.

Supplement: Figure 2—figure supplement 1—source data 1. [file elife-70948-fig2-figsupp1-data1.pdf]

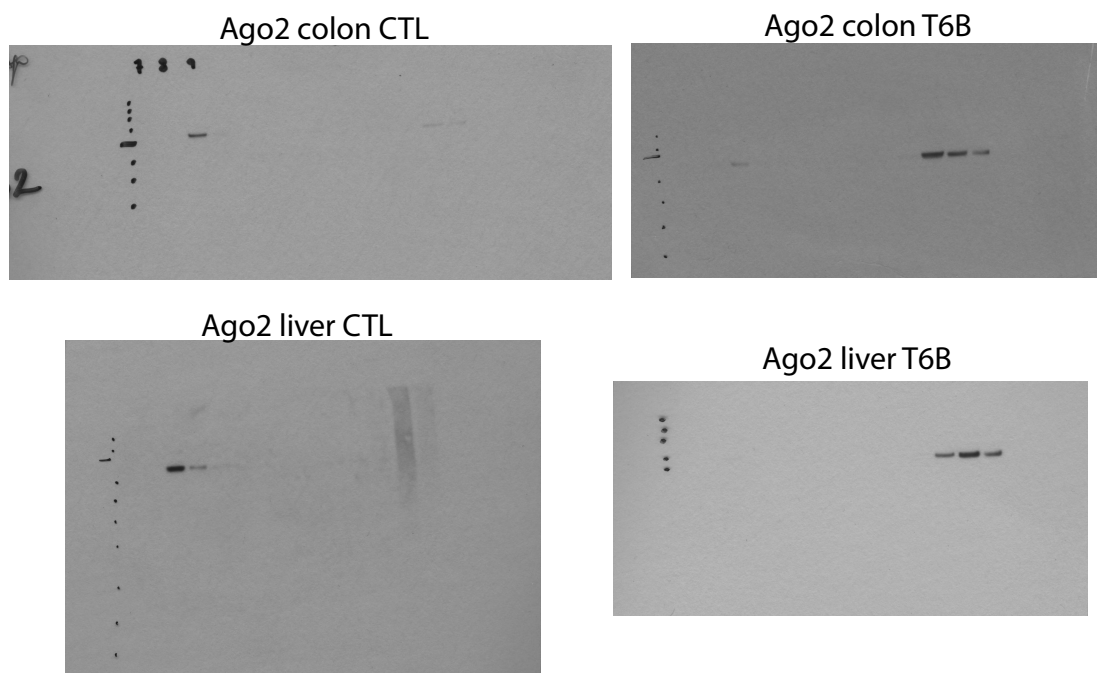

Figure 2-supporting figure 4-source data 1.Unedited blots shown in Figure 2-figure supplement 4.

Supplement: Figure 2—figure supplement 4—source data 1. [file elife-70948-fig2-figsupp4-data1.pdf]
