# Supplementary material for: Inducible and reversible inhibition of miRNA-mediated gene repression in vivo
Source: eLife. 2021 Aug 31;10:e70948. doi: 10.7554/eLife.70948 (PMC8476124; doi:10.7554/eLife.70948)
Supplement: Figure 1—source data 5. [file elife-70948-fig1-data5.pdf]

Top: Ago2 no dox
bottom: Ago2 T6B on dox

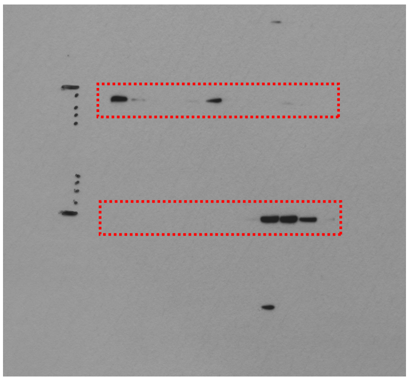

Top: tnrc6 T6B no dox
bottom: tnrc6 T6B on dox

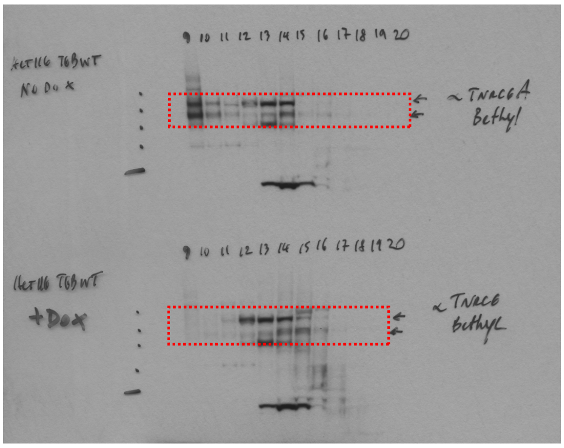

Ago2 T6B-mut on dox

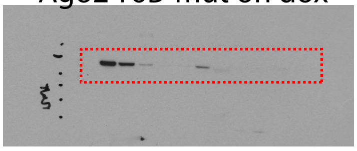

PABP1 T6B mut on dox

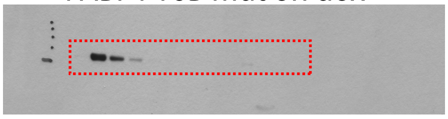

tnrc6 T6B mut on dox

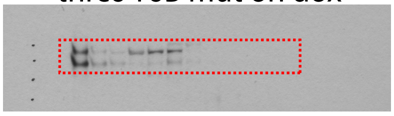

Top: T6B T6B-mut on dox
bottom: T6B T6B on dox

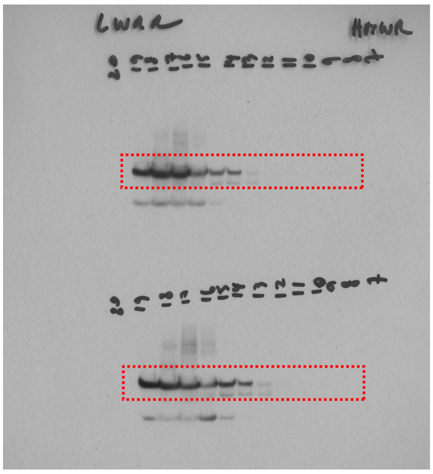

Top: PABP1 T6B no dox
bottom: PABP1 T6B on dox

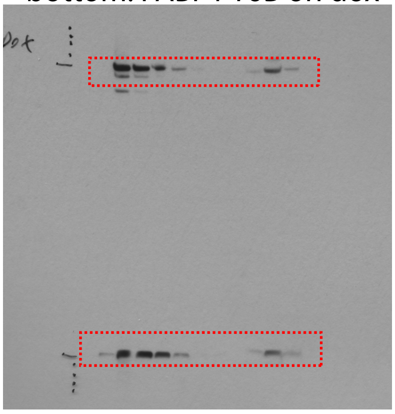

Figure 1-source data 5. Uncropped gels used in Figure 1C. Red dashed boxes indicate the cropped area used in figure.
