# Supplementary material for: Inducible and reversible inhibition of miRNA-mediated gene repression in vivo
Source: eLife. 2021 Aug 31;10:e70948. doi: 10.7554/eLife.70948 (PMC8476124; doi:10.7554/eLife.70948)
Supplement: Figure 1—source data 7. [file elife-70948-fig1-data7.pdf]

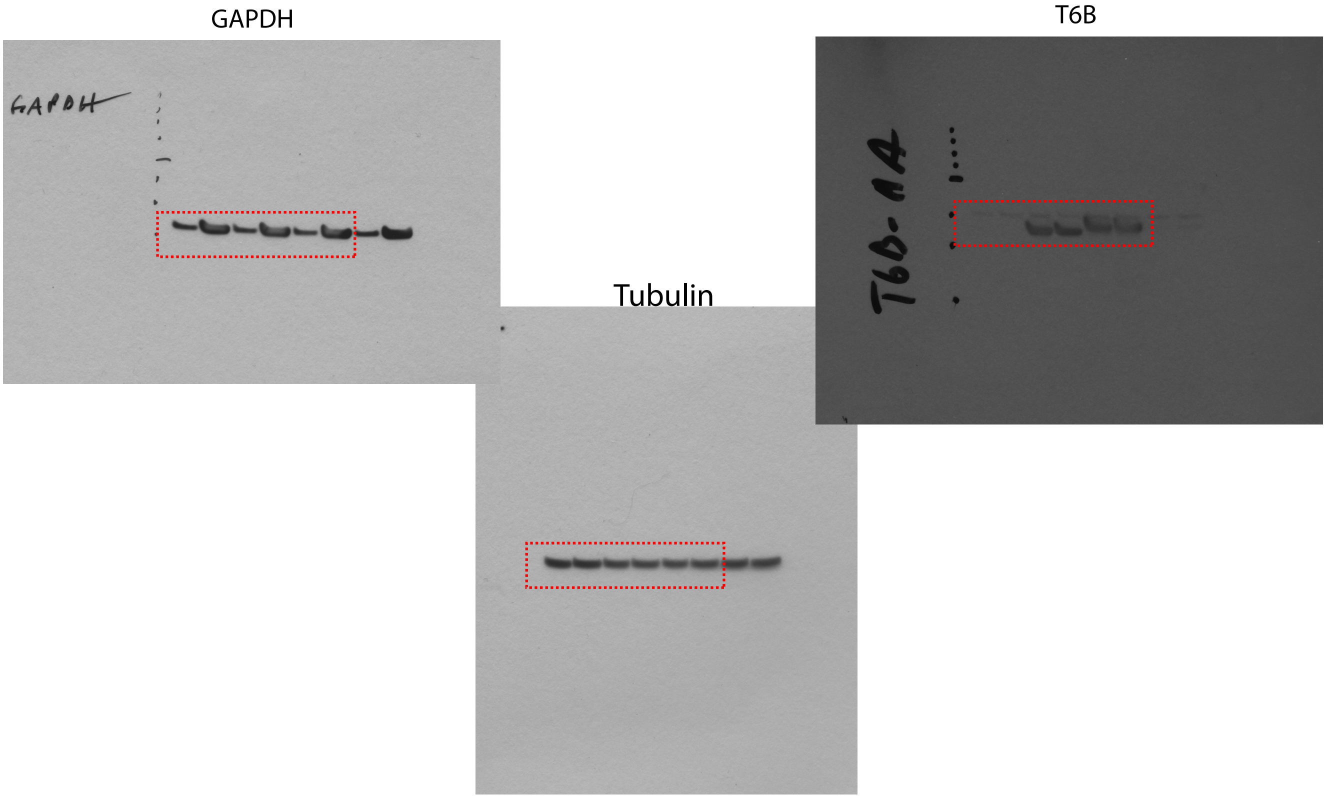

Figure 1-source data 7. Uncropped gels used in Figure 1F. Red dashed boxes indicate the cropped area used in figure.
