# Supplementary material for: Inducible and reversible inhibition of miRNA-mediated gene repression in vivo
Source: eLife. 2021 Aug 31;10:e70948. doi: 10.7554/eLife.70948 (PMC8476124; doi:10.7554/eLife.70948)
Supplement: Figure 1—figure supplement 2—source data 1. [file elife-70948-fig1-figsupp2-data1.pdf]

Ago in MEF-T6B

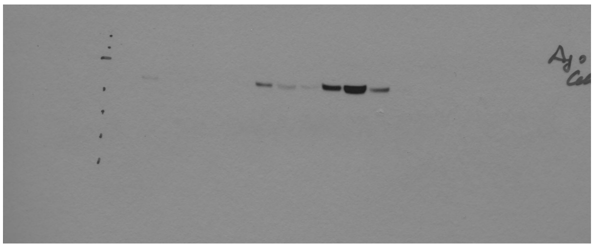

Ago in MEF-T6B-mut

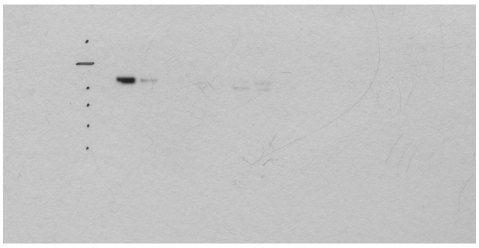

T6B in MEF-T6B

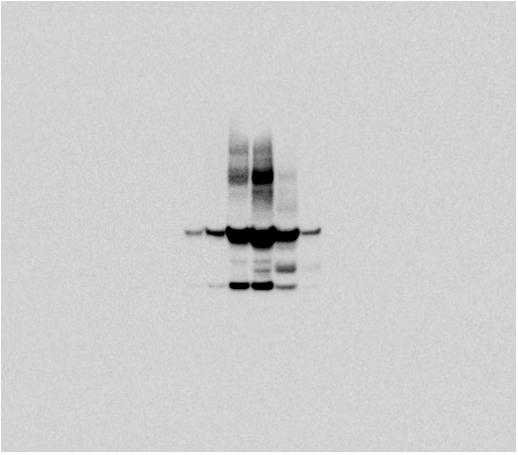

T6B in MEF-T6B-mut

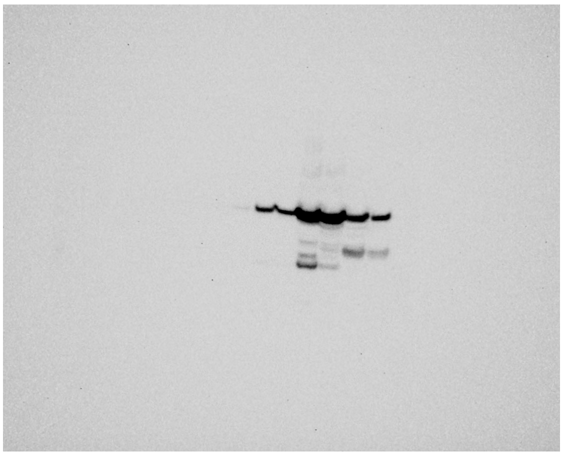

Figure 1-figure supplement 2-source data 1. Unedited blots shown in Figure 1-figure supplement 2.
