# Supplementary material for: Inducible and reversible inhibition of miRNA-mediated gene repression in vivo
Source: eLife. 2021 Aug 31;10:e70948. doi: 10.7554/eLife.70948 (PMC8476124; doi:10.7554/eLife.70948)
Supplement: Figure 1—figure supplement 2—source data 2. [file elife-70948-fig1-figsupp2-data2.pdf]

Ago in MEF-T6B

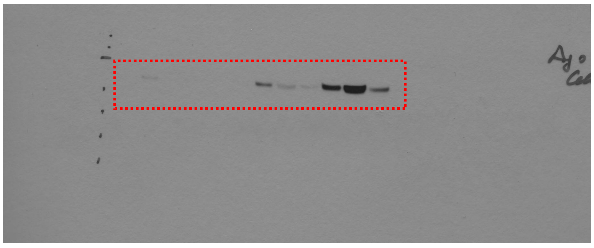

Ago in MEF-T6B-mut

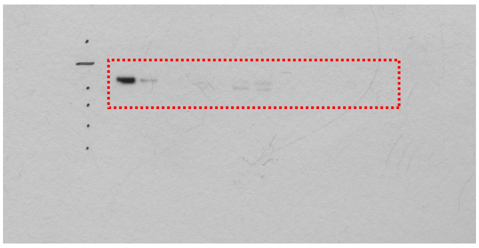

T6B in MEF-T6B

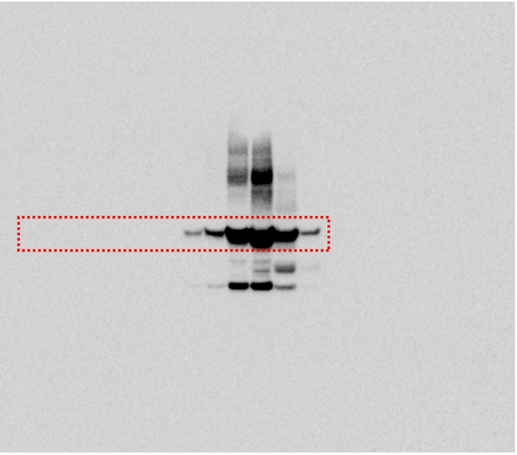

T6B in MEF-T6B-mut

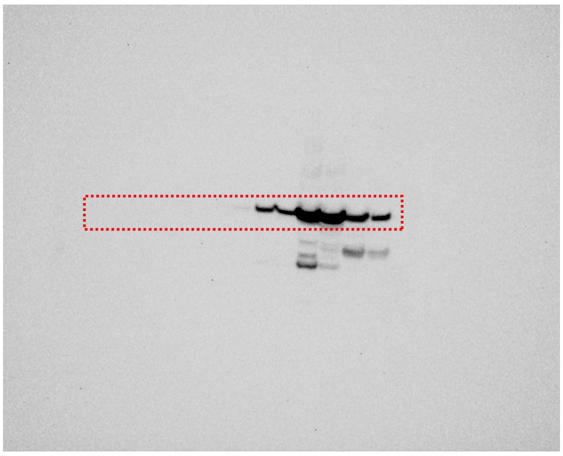

Figure 1-figure supplement 2-source data 2. Uncropped blots shown in Figure 1-figure supplement 2. Red dashed boxes indicate the cropped area used in figure.
