# Supplementary material for: Inducible and reversible inhibition of miRNA-mediated gene repression in vivo
Source: eLife. 2021 Aug 31;10:e70948. doi: 10.7554/eLife.70948 (PMC8476124; doi:10.7554/eLife.70948)
Supplement: Figure 2—source data 4. [file elife-70948-fig2-data4.pdf]

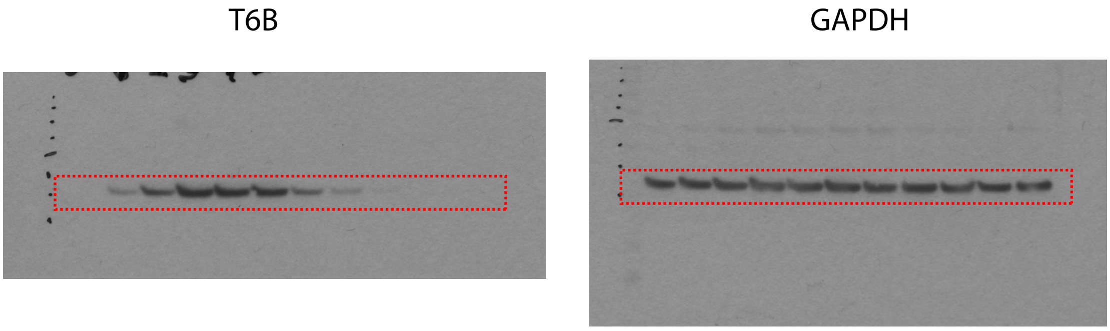

Figure 2-source data 4. Uncropped blots shown in Figure 2C. Red dashed boxes indicate the cropped area used in figure.
