# Supplementary material for: Inducible and reversible inhibition of miRNA-mediated gene repression in vivo
Source: eLife. 2021 Aug 31;10:e70948. doi: 10.7554/eLife.70948 (PMC8476124; doi:10.7554/eLife.70948)
Supplement: Figure 2—source data 6. [file elife-70948-fig2-data6.pdf]

AGO2

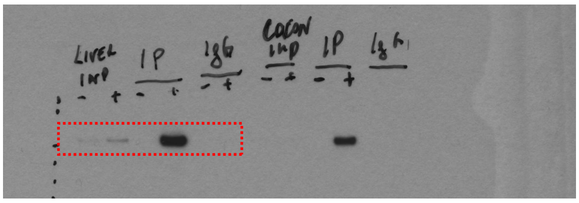

T6B

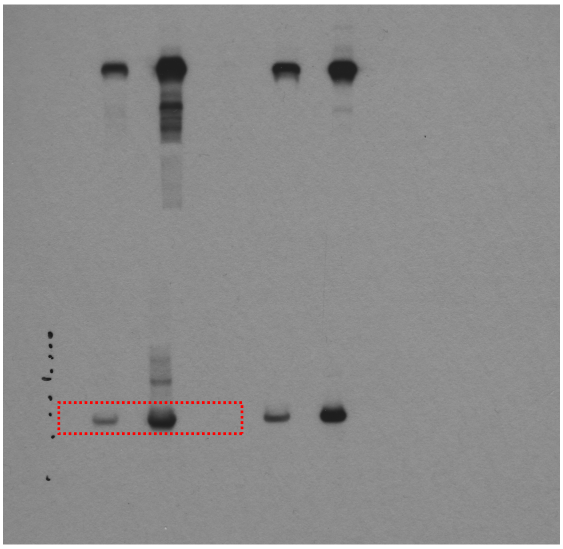

GAPDH

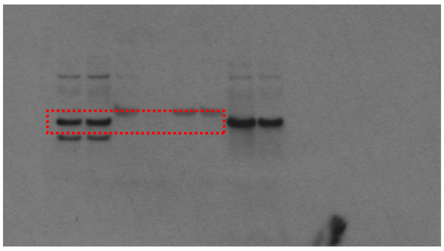

Figure 2-source data 6. Uncropped blots shown in Figure 2D.Red dashed boxes indicate the cropped area used in figure.
