# Supplementary material for: Inducible and reversible inhibition of miRNA-mediated gene repression in vivo
Source: eLife. 2021 Aug 31;10:e70948. doi: 10.7554/eLife.70948 (PMC8476124; doi:10.7554/eLife.70948)
Supplement: Figure 2—source data 8. [file elife-70948-fig2-data8.pdf]

Ago2 day 0

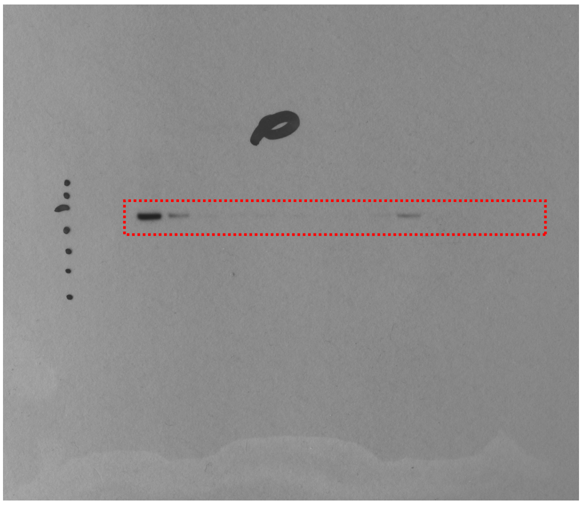

Ago2 day 5

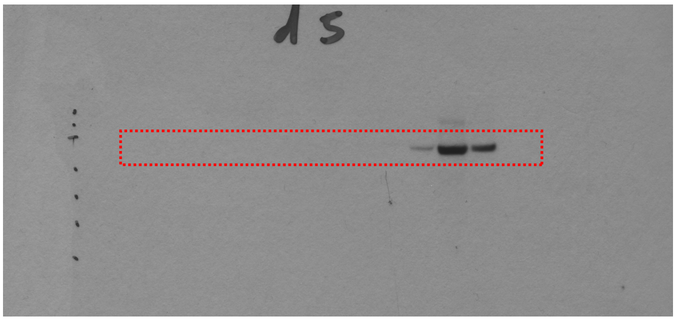

Ago2 day 5 off dox

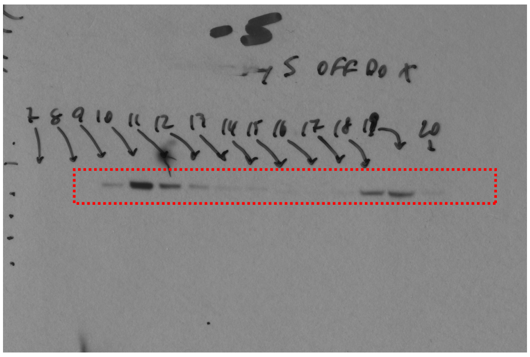

Figure 2-source data 8. Uncropped blots shown in figure 2E. Red dashed boxes indicate the cropped area used in figure.
