# Supplementary material for: Inducible and reversible inhibition of miRNA-mediated gene repression in vivo
Source: eLife. 2021 Aug 31;10:e70948. doi: 10.7554/eLife.70948 (PMC8476124; doi:10.7554/eLife.70948)
Supplement: Figure 2—figure supplement 3—source data 1. [file elife-70948-fig2-figsupp3-data1.pdf]

T6B

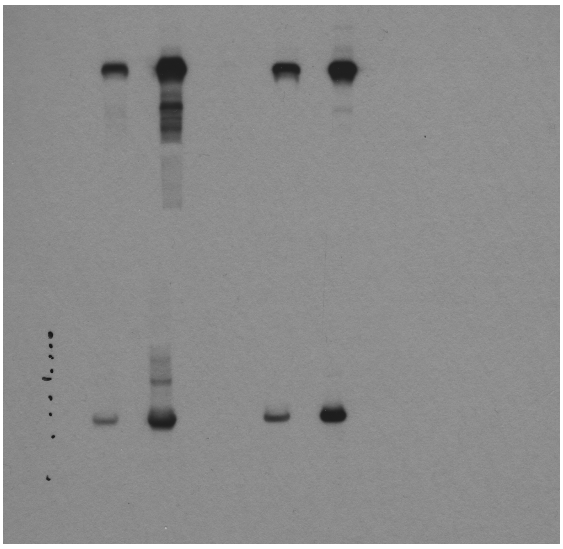

AGO2

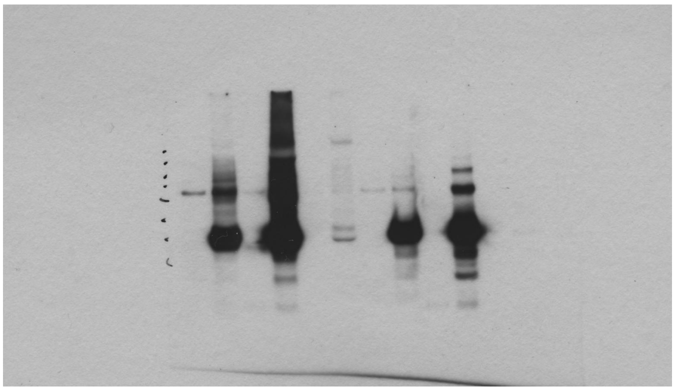

GAPDH

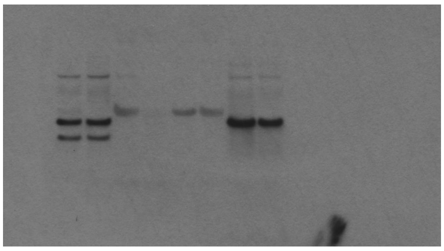

Figure 2-figure supplement 3-source data 1. Unedted blots shown in Figure 2-figure supplement 3
