# Supplementary material for: Inducible and reversible inhibition of miRNA-mediated gene repression in vivo
Source: eLife. 2021 Aug 31;10:e70948. doi: 10.7554/eLife.70948 (PMC8476124; doi:10.7554/eLife.70948)
Supplement: Figure 3—source data 1. [file elife-70948-fig3-data1.pdf]

		CTRL (n=5)	T6B (n=5)	*P* value
RBC	$10^6/\mu L$	10.8 ± 0.483	10.7 ± 0.292	0.9225
HGB	g/dL	16.4 ± 0.589	14.8 ± 0.277	0.0052
HCT	%	57.0 ± 1.74	52.0 ± 0.631	0.0037
MCV	fL	52.8 ± 1.33	48.6 ± 0.858	0.0041
MCH	pg	15.2 ± 0.2	13.9 ± 0.195	0.0002
RDW	%	22.8 ± 0.342	25.5 ± 0.716	0.0019
RET	$10^3/\mu L$	564 ± 68.5	569 ± 76.5	0.9273
WBC	$10^3/\mu L$	7.46 ± 1.16	9.16 ± 1.23	0.2608
PLT	$10^3/\mu L$	990 ± 290	1270 ± 154	0.2728

Figure 3-source data 1: Complete blood counts (CBCs) of whole blood from $R26^{T6B}$ and $R26^{CTL}$ mice taken after 3 weeks on doxycycline. Abbreviations are as follows: RBC: red blood cell count; HGB: hemoglobin; HCT: hematocrit; MCV: mean corpuscular volume; MCH: mean cell hemoglobin; RDW: red cell distribution width; RET: reticulocyte count; WBC: white blood cell count; PLT: platelet count.
